# Surgical Treatment of Severe Idiopathic Flexible Flatfoot by Evans–Mosca Technique in Adolescent Patients: A Long-Term Follow-Up Study

**DOI:** 10.1155/2021/8843091

**Published:** 2021-01-20

**Authors:** Vincenzo De Luna, Fernando De Maio, Alessandro Caterini, Martina Marsiolo, Lidio Petrungaro, Ernesto Ippolito, Pasquale Farsetti

**Affiliations:** Department of Clinical Sciences and Traslational Medicine, Division of Orthopaedic Surgery, University of “Tor Vergata”, Rome, Italy

## Abstract

Flexible idiopathic flatfoot is very common in growing age and rarely causes pain or disability. Surgery is indicated only in severe symptomatic cases that are resistant to conservative treatment, and numerous surgical procedures have been proposed. Lateral column calcaneal lengthening as described by Evans and modified by Mosca is a widely used surgical technique for the correction of severe symptomatic flexible flatfoot. In the present study, we report the long-term clinical and radiographic results in 14 adolescent patients (mean age: 12.8 years) affected by severe symptomatic flexible flatfoot, surgically treated by Evans–Mosca procedure, for a total of 26 treated feet (12 cases bilateral and 2 unilateral). In all cases, surgery was indicated for the presence of significant symptoms resistant to nonsurgical management. Clinical evaluation was made according to the American Orthopedic Foot and Ankle Society (AOFAS) Ankle-Hindfoot Scale, the Foot and Ankle Disability Index (FADI) Score, and Yoo et al.'s criteria. Radiographic evaluation was made using anteroposterior and lateral weight-bearing radiographs of the feet to evaluate Meary's angle and Costa–Bertani's angle and to evaluate possible osteoarthritic changes in the midtarsal joints. At follow-up (mean: 7 years and 7 months), we observed a satisfactory result in all patients. The mean average score of the AOFAS Ankle-Hindfoot Scale improved from 60.03 points to 95.26; the mean FADI score improved from 71.41 to 97.44; and according to Yoo et al.'s criteria, the average clinical outcome score was 10.96. At radiographic examination, nonunion of the calcaneal osteotomy was never observed. Meary's angle improved from an average preoperative value of 25° to 1.38° at follow-up; Costa–Bertani's angle improved from an average preoperative value of 154.2° to 130.9° at follow-up. In no case, significant radiographic signs of midtarsal joint arthritis were observed. According to our results, we believe that Evans–Mosca technique is a valid option of surgical treatment for severe idiopathic flexible flatfoot and allows a satisfactory correction of the deformity with a low rate of complications.

## 1. Introduction

Flexible flatfoot is a very common foot condition observed during skeletal growth, characterized by a depression of the medial arch with an associated hindfoot valgus and forefoot abduction [1–3]; also, a short Achilles tendon is often present [4]. Flexible flatfoot rarely causes pain or disability in growing age patients and generally does not require treatment because in the majority of cases, the medial arch spontaneously elevates during the first decade of life. Moreover, it is well known that many adults have a flexible flatfoot without any significant limitation in activities of daily living or sport activities [5]. Surgery is indicated only in severe symptomatic cases with diffuse activity-related pain and medial foot calluses, observed usually in adolescents, in which conservative treatment has failed [5, 6]. The correct indication, time, and methods of surgical treatment for the correction of severe symptomatic flatfoot are however still debated, and several surgical procedures have been proposed [7–11]. The most common operations performed are arthroereisis, lateral calcaneal lengthening osteotomy, and triple arthrodesis [8–11]. Soft tissue surgical procedures have also been reported, but when they are performed alone, they lead to unsatisfactory results [5]. Evans, more than forty years ago [10], introduced the lateral column lengthening for the treatment of severe symptomatic flatfoot and proposed a calcaneal lengthening osteotomy for its correction. More recently, Mosca elaborated a modification of this technique, proposing an opening wedge osteotomy with a trapezoidal, tricortical iliac crest wedge [11].

The aim of the present study was to report the long-term results obtained in a series of 14 adolescents affected by severe symptomatic flexible flatfoot, surgically treated by Evans–Mosca technique associated to a soft tissue procedure of the medial side of the foot and, in almost 50% of cases, percutaneous lengthening of the Achilles tendon.

## 2. Materials and Methods

We reviewed 14 patients affected by severe idiopathic symptomatic flexible flatfoot, surgically treated by lateral column lengthening according to Evans–Mosca procedure associated to the tibialis posterior tendon and talonavicular joint capsule strain and, in some cases, percutaneous lengthening of the Achilles tendon. Eight patients were male, and 6 were female. The mean age of the patients at surgery was 12.8 years (range: from 11 to 14.6 years); 12 cases were operated bilaterally, for a total of 26 feet. In all our patients treated bilaterally, we performed the two procedures at different times, with a distance between the two operations that ranged from 8 months to one year.

**Figure 1:**
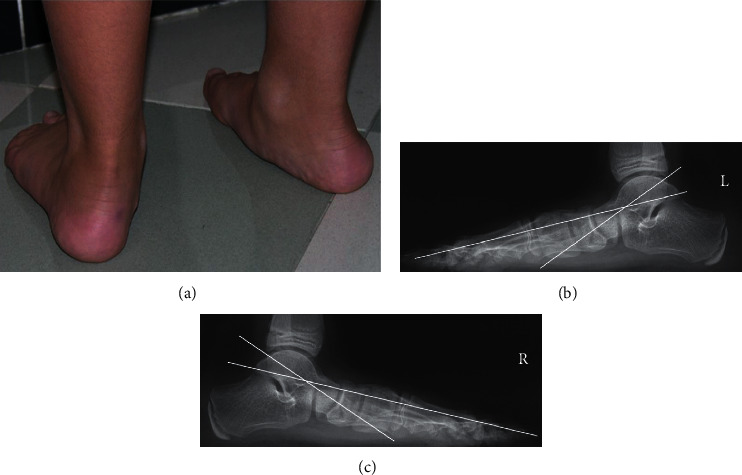
Clinical aspect of an 11-year-old boy with bilateral painful idiopathic flexible flatfoot (a). At radiographic examination, Meary's angle measured 26° of the left foot (b) and 23° of the right foot (c). The patient was treated conservatively by an insert sole, but this treatment failed, and a surgical procedure was proposed.

Regarding the clinical evaluation before surgery, all patients had a medial longitudinal arch abnormally depressed or absent with a normal subtalar joint mobility. In 12 feet, a short Achilles tendon was also present with an associated limitation of the ankle dorsiflexion. Preoperatively, standard weight-bearing radiographs of the foot in anteroposterior and lateral projections confirmed the presence of a flexible flatfoot in all cases. Conservative treatment aims to improve or solve foot pain without changing the foot shape. In our series, it was based on strengthening exercises, custom orthosis, or corrective shoes, but failed in all patients (Figure 1). The surgical technique was performed under general anesthesia with a thigh tourniquet. The incision was made with an oblique direction over the sinus tarsi towards the inferior border of the calcaneus. The calcaneus was exposed, and the osteotomy was performed approximately 1.5 cm proximally from the calcaneocuboid joint, between the anterior and middle facets of the subtalar joint. The osteotomy should be oriented from posterolateral to anteromedial to avoid damage of the middle facet. After the distraction osteotomy was performed with a spreader, a tricortical iliac crest autologous bone graft, taken from the same side of the operated side, was inserted into the space to obtain a lengthening of the lateral column through the anterior part of the calcaneus. The osteotomy was stabilized with one or two Kirschner or Steinmann wires, according to the age of the patients and the size of the foot. A second incision was made along the medial border of the foot. The tibialis posterior was strained through a tendon release, and the talonavicular joint capsule, including the spring ligament, was incised and plicated plantarmedially. In 12 feet, in which a short Achilles tendon was present, percutaneous lengthening was also performed. After surgery, a nonweight bearing below the knee cast was applied for 6–8 weeks. Weight bearing was permitted only after radiographic healing of the osteotomy was observed.

All patients were evaluated clinically, analyzing the preoperative function of the foot in comparison to the follow-up examination, using the American Orthopedic Foot and Ankle Society (AOFAS) Ankle-Hindfoot Scale [12], the Foot and Ankle Disability Index (FADI) Score [13], and, only at follow-up, Yoo et al. criteria [14]. From a radiographic point of view, all patients were evaluated by an anteroposterior and lateral weight-bearing examination of both feet, taken before surgery and at follow-up, to measure both Meary's and Costa–Bertani's angles.

**Figure 2:**
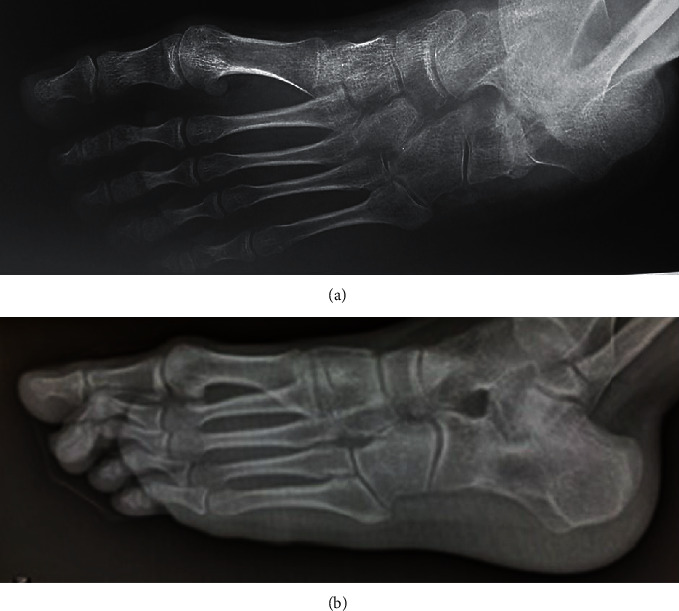
Same patient of Figure 1, three years later. The patient was surgically treated by Evans–Mosca procedure bilaterally with an interval of six months. The radiographic examination of the left foot, performed in oblique view 3 months after surgery, showed good lengthening of the calcaneus with the presence of the tricortical bone graft (a). At an intermediate follow-up, 7 years later, the graft was perfectly remodeled in the calcaneal bone with the maintenance of calcaneal lengthening and without any sign of osteoarthritis of the calcaneocudoid joint (b).

Moreover, at follow-up radiographic examination, we looked for possible subluxation or osteoarthritic changes of the calcaneocuboid or talonavicular joints as well as the remodeling of the tricortical bone graft into the calcaneal osteotomy (Figure 2).

Statistical analysis was performed using paired Student's *t*-test, which was considered significant when *p* value measured <0.05.

## 3. Results

All patients were reviewed after a mean follow-up of 7 years and 7 months (from 6 y and 5 m to 12 y).

According to the AOFAS system [12], the average preoperative score was 69.03 points (from 59 to 78 points), while the average score at follow-up improved to 95.26 (from 88 to 100). According to the FADI method [13], the average preoperative score was 71.41 (from 59.5 to 75.3), while the average score at follow-up was 97.44 (from 88.5 to 100). The difference between the preoperative and final score evaluated at follow-up was statistically significant for both clinical rating scales adopted (*p* <0.01). According to Yoo et al.'s criteria [14], the average clinical outcome score at follow-up was 10.96 (from 8 to 12); since a score equal to or greater than 8 corresponds to a satisfactory clinical result, we obtained a satisfactory clinical result in all patients. We found no difference in terms of clinical results and satisfaction between patients treated on one foot or both feet (Table 1).

**Figure 3:**
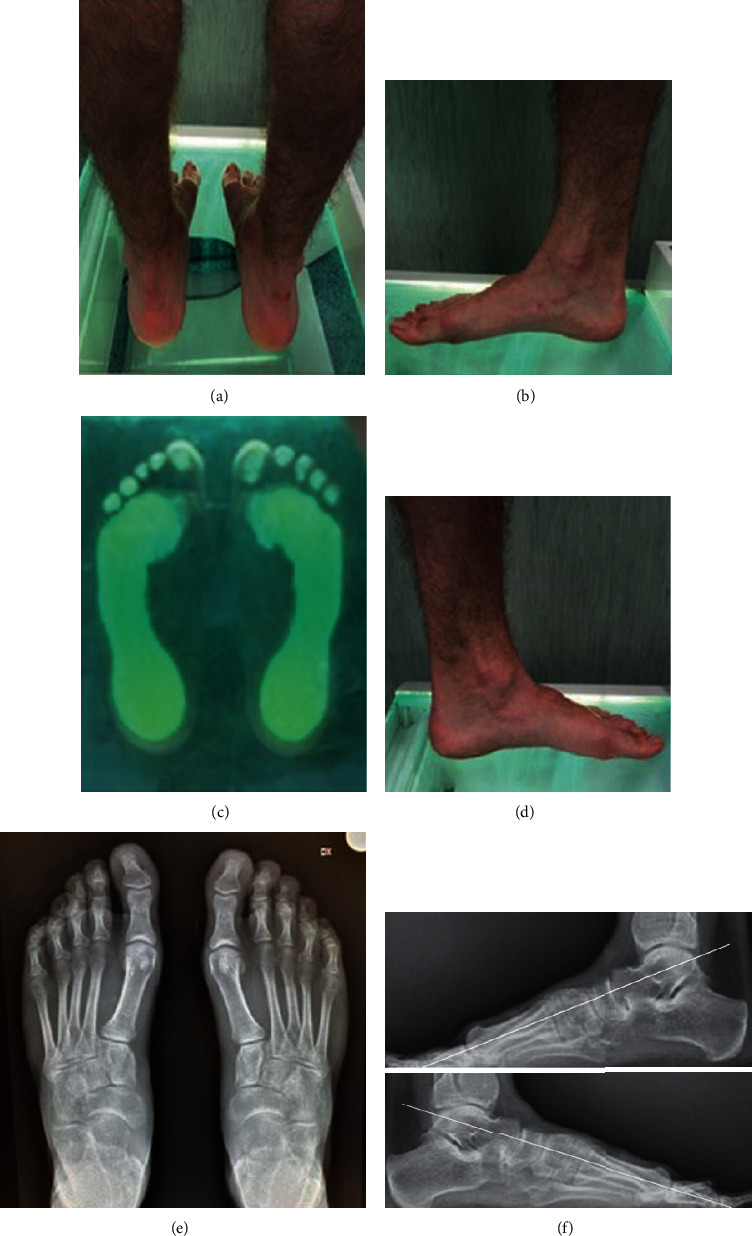
Same patient of Figure 1, at the final follow-up, 11 years after surgery. The patient was pain free. Clinical and podoscopic aspects of both feet showed an excellent result ((a)–(d)). At radiographic examination, Meary's angle was normal bilaterally (0°), in the absence of midtarsal joint osteoarthritis ((e) and (f)).

At radiographic examination, nonunion of the calcaneal osteotomy was never observed, and the tricortical bone graft was always remodeled. Meary's angle improved from an average preoperative value of 25° (from 22° to 32°) to an average value of 1.4° (from 0° to 4°) at follow-up. Costa–Bertani's angle improved from an average preoperative value of 154.2° (from 150° to 160°) to an average value of 130.9° (from 124° to 138°) at follow-up. Also, for both radiographic parameters, the difference between preoperative and final angle values was statistically significant (*p* <0.01). Calcaneocuboid joint subluxation was observed in only 3 feet (11.5%), while significant calcaneocuboid or talonavicular joint osteoarthritis was never observed (Figure 3).

## 4. Discussion

Flexible idiopathic flatfoot is very common in children and rarely causes pain or disability. On the contrary, congenital flatfoot caused by tarsal coalition, Marfan syndrome, or other congenital disorders, is usually painful, but uncommon in comparison to other congenital, pediatric, orthopedic diseases [15–18]. However, in some cases, especially in adolescents, flexible flatfoot may cause pain and disability, and surgery may be indicated when conservative treatment fails [19].

In severe idiopathic symptomatic adolescent flatfoot, surgical indication and the type of surgical treatment to perform are still controversial. Arthroereisis of the subtalar joint for the correction of severe flexible flatfoot in growing children is a widely used technique [20, 21]. This surgical procedure restricts excessive subtalar joint eversion by placing a synthetic implant. Different devices have been proposed over time including bioabsorbable implants. Good results have been reported in the literature for the correction of flexible flatfoot in growing children with arthroereisis of the subtalar joint [9, 20, 22], but pain at the level of the sinus tarsi is a possible complication of these techniques, and a second surgery for implant removal may be required [5, 23, 24]. For this reason, we preferred Evans–Mosca surgical procedures instead of arthroereisis in our series of adolescent patients.

Evans [10] believed that the lateral column in flatfeet was shorter than the medial column and first proposed a calcaneal lengthening osteotomy for the correction of valgus deformity, without an opening wedge osteotomy. This concept was elaborated by Mosca [5, 11] that published a modified technique utilizing a trapezoidal, tricortical iliac crest wedge to perform both an opening wedge and a distracting osteotomy and reported correction of all components of the deformity.

The calcaneal lengthening osteotomy for the surgical treatment of severe symptomatic idiopathic flexible flatfoot was later used by other authors with satisfactory results. Dogan et al. [25], in a series of 13 patients (25 feet) treated for flexible pes planovalgus by calcaneal lengthening osteotomy, reported that foot pain was eliminated in all patients but one. They concluded that the advantages of this procedure are the preservation of subtalar joint motion and the correction of the deformity in multiple plans. Moraleda et al. [26] also reported good clinical and radiographic results in a series of 21 children (33 feet) with symptomatic flexible flatfoot surgical treatment with a calcaneal lengthening osteotomy. More recently, Kumar and Sonanis [27] reported a systematic review on lateral column lengthening performed in adolescent pes planovalgus deformity. These authors identified seven studies with 103 patients involving 156 feet and concluded that lateral column lengthening leads to good clinical and radiological outcome with high patient satisfaction and acceptable complication rate. They also concluded that the literature is mostly retrospective, and there is need for other studies.

The indication of lateral calcaneal lengthening osteotomy for idiopathic flatfoot in children has been extended to other forms of flatfoot with a low incidence of complications [26]. Marengo et al. [28] reported the clinical and radiological outcome of calcaneal lengthening osteotomy for flatfoot deformity of various etiologies in 27 skeletally immature patients (38 feet). Clinical outcome was satisfactory in 89% of cases, and all radiographic parameters improved significantly. They concluded that calcaneal lengthening osteotomy is not contraindicated in symptomatic flatfoot of different etiologies, except neuromuscular disease-related flatfoot that can affect bone quality and reduce foot flexibility. They also reported that calcaneocuboid joint subluxation is frequently observed but has little functional impact as it tends to remodel over time. Similar to other CT studies that analyzed the results of treatment of the congenital clubfoot [29], Canavese et al. [30] published a study on postoperative CT-scan 3D reconstruction of the calcaneus following lateral calcaneal lengthening osteotomy performed in 14 children (20 feet) affected by symptomatic flatfoot with different etiologies. This study showed that subtalar anatomy presented significant anatomical variations among these examined patients; however, clinical evaluation at follow-up showed satisfactory outcome in 80% of cases. Calcaneal lengthening for flatfoot deformity in patients with cerebral palsy has also been reported with good results [31]. Andreacchio et al. [32] concluded that calcaneal lengthening is a successful treatment for flexible planovalgus foot deformity in ambulatory children with spastic CP. Regarding other etiologies, Mosca and Bevan [33] reported good results for correcting deformity and relieving pain in rigid flatfeet of 8 patients (13 feet), affected by talocalcaneal tarsal coalition, treated by calcaneal lengthening osteotomy with gastrocnemius or Achilles tendon lengthening. Guha and Perera [34] reported that calcaneal lengthening osteotomy may be performed even in the correction of adult flexible flatfoot, but they concluded that an essential prerequisite for using this technique is the absence of arthritis of the subtalar joint.

Our long-term results in a group of adolescents surgically treated for severe symptomatic idiopathic flexible flatfoot by the Evans–Mosca technique, in association with a strain of the tibialis posterior tendon and joint capsule, confirmed the good results reported by the aforementioned studies. All our patients were satisfied with the final result and referred a significant improvement of the preoperative symptoms.

In our patients, we paid particular attention to performing the calcaneal osteotomy into the interval between the anterior and middle facets of the subtalar joint as suggested by Ragab et al. [35], and at follow-up, we never observed significant radiographic changes of the midtarsal joint as reported by some studies [36, 37]. We always used a tricortical iliac crest autologous bone graft inserted into the osteotomy, as described by Mosca [11], to obtain lengthening of the lateral column of the calcaneus and never observed complications in terms of nonunion or loss of correction. Also, we never observed any patient morbidity from graft harvesting at the level of the iliac crest; however, some authors reported satisfactory results using autogenous bone graft from a different donor site or allogenic bone graft [38–40]. We had no personal experiences with titanium wedge insertion. We prefer to use bone autograft to obtain a better and faster integration in the native bone and to better maintain the correction obtained. However, to avoid the risk of pain at the level of the donor site, the use of allograft might be considered.

## 5. Conclusion

According to our long-term results, we believe that Evans–Mosca surgical procedure is an effective solution for the treatment of idiopathic symptomatic flexible flatfoot resistant to conservative treatment and allows the maintenance of the surgical correction without the development of osteoarthritic changes.

## Figures and Tables

**Table 1:** Preoperative and final (at follow-up) clinical and radiographic results in 26 flatfeet surgically treated by Evans–Mosca technique.

AOFAS (preop)	69.03 (59–79)	*p* value <0.01
AOFAS (follow up)	95.26 (88–100)	

FADI (preop)	71.41 (59.5–75.3)	*p* value <0.01
FADI (follow up)	97.44 (88.5–100)	

Yoo et al. (follow up)	10.96 (8–12)	—

Meary's angle (preop)	25° (22°–32°)	*p* value <0.01
Meary's angle (follow up)	1.4° (0°–4°)	

Costa–Bertani angle (preop)	154.2° (150°–160°)	*p* value <0.01
Costa–Bertani angle (follow up)	130.9° (124°–138°)	

## Data Availability

The data used to support the findings of this study are available from the corresponding author upon reasonable request.
